# The Asthma-associated PER1-like domain-containing protein 1 (PERLD1) Haplotype Influences Soluble Glycosylphosphatidylinositol Anchor Protein (sGPI-AP) Levels in Serum and Immune Cell Proliferation

**DOI:** 10.1038/s41598-020-57592-9

**Published:** 2020-01-20

**Authors:** Yang Yie Sio, Ramani Anantharaman, Sean Qiu En Lee, Sri Anusha Matta, Yu Ting Ng, Fook Tim Chew

**Affiliations:** Department of Biological Sciences, National University of Singapore, Singapore, Singapore

**Keywords:** Functional genomics, Haplotypes, Genetic association study

## Abstract

Post-glycosylphosphatidylinositol (GPI) attachment to proteins 3, also known as PGAP3 or PERLD1 (PER1-like domain-containing protein 1), participates in the lipid remodeling process of glycosylphosphatidylinositol (GPI) anchor proteins during post-translational modification. Functional defect in PERLD1 was previously hypothesized to influence this process in T-cells and their subsequent activation and proliferation. This current study aims to functionally characterize PERLD1 genetic variants and relate this with human immune cells proliferation rate upon stimulation. We first showed the association between a PERLD1 tag-single nucleotide polymorphism (tagSNP), rs2941504, and the development of asthma in our study population. This association remained significant after conditioning for the other asthma-associated SNP rs8076131 that is also located within the 17q12–21 region. Subsequent sequencing of 40 unrelated Singapore Chinese individuals identified 12 more common PERLD1 SNPs (minor allele frequency > 5%) that are in linkage disequilibrium (LD, r^2^ > 0.8) with rs2941504. Through *in vitro* studies, 7 of these SNPs were found to form a functional haplotype that influences alternative splicing of PERLD1 transcript. This result was validated in human peripheral blood mononuclear cell (PBMC), where the minor haplotype (Hap2) was shown to be associated with significantly increased PERLD1 truncated transcript. Additionally, Hap2 was found to be related to increased levels of several soluble GPI-anchored proteins (such as sCD55 and sCD59) in serum. Elevated sCD55 in the serum was demonstrated to reduce the proliferation rate of PBMCs upon phytohaemagglutinin (PHA) stimulation. Taken together, the current study has shown a functional PERLD1 haplotype, which modifies PBMC sensitivity upon stimulation and may contribute to the individual’s susceptibility to allergic asthma.

## Introduction

Asthma is a chronic respiratory disease affecting more than 300 million people worldwide^1^. This disease is characterized by chronic inflammation in the lung airways and suggested to be mediated by T helper 2 (Th2) cells in the immune system^2,3^. In asthmatic patients, increased responsiveness to innate type 2 mediators was observed in allergen-specific Th2 cells^4^. However, the underlying mechanism leading to this aggressiveness remains unknown.

Based on previous study on its yeast homolog PER1 protein, post- glycosylphosphatidylinositol (GPI) attachment to proteins 3 (PGAP3 or PERLD1) was suggested to be involved in the lipid remodeling process of GPI anchors^5^. GPI anchors are glycolipid moieties added to a specific group of GPI-anchored proteins (GPI-AP) during post-translational modification. After the addition of GPI anchor, the inositol-linked side chain of this glycolipid moiety would be removed by post-GPI attachment to proteins 1 (PGAP1), and GPI-AP is then transferred to the Golgi apparatus^6^. Subsequently PERLD1, also termed PGAP3, would remove unsaturated fatty-acid side chains of GPI anchor at the sn2 position^6^. This empty sn2 position is then replaced by stearic acid, catalyzed by PGAP2 protein^6^. In overall, GPI anchors modification was regarded as crucial for the transportation of GPI-AP from ER to Golgi and their subsequent association with lipid rafts of cell membrane^5^ or released as soluble fractions in the serum.

GPI-APs are expressed in multiple tissue types, including immune cells. Cross-linking of GPI-APs, such as CD48 and CD59, could induce proliferation of T lymphocytes^7^. It has also been reported that CD48 would participate in T-cell receptor activation and several other inflammatory pathways that lead to asthma pathogenesis^8,9^. CD59 was shown to modulate T-cell activation by triggering multiple signaling pathways, including ZAP-70 dependent and independent pathways^10^. Furthermore, the GPI-anchored CD55 protein was reported to inhibit the complement signaling pathway, which could lead to the suppression of T cell immunity^11,12^. Independent of this complement pathway, direct stimulation of CD55 was also found capable of activating human CD4+ T cell^13^. Therefore, the involvement of these GPI-APs in regulating the proliferative ability of T cells might suggest the potential role of PERLD1 in contributing to the inflammatory phenotype of allergic asthma.

In a pooled Genome-wide Association Study (GWAS) previously conducted by our group in the Singapore Chinese population, PERLD1 tag-single nucleotide polymorphism (tag-SNP) rs2941504 was shown to be significantly associated with the susceptibility to allergic asthma^14^. This tag-SNP was also associated with early-onset asthma in a combined cohort comprises of subjects from European and Korean populations^15^. Besides, prior studies have found significant expression quantitative trait locus (eQTL) of rs2941504 with GSDMB and ORMDL3 genes^15–17^. Nevertheless, no study has thus far characterized its functional implications on the PERLD1 gene or associated this SNP with the pathogenesis of asthma. Here, we report PERLD1 tag-SNP rs2941504 as part of a functional haplotype that is linked to asthma susceptibility through influencing immune cell proliferative capabilities. Multiple characteristics of peripheral blood mononuclear cell (PBMC), including PERLD1 transcript expression profile, cell surface and secreted GPI-AP levels, and proliferation upon phytohaemagglutinin (PHA) stimulation were assessed with haplotypes formed by tag SNP rs2941504 and its associated SNPs within the PERLD1 region. Collectively, these findings would provide a mechanism that links PERLD1 to the inflammatory aspect of allergic asthma.

## Results

### Genotyping of the asthma-associated SNPs

Tag-SNP rs2941504 has previously been identified as an asthma-associated signal in Singapore Chinese. We currently showed this asthma-associated signal as an independent signal from another widely reported asthma-associated SNP rs8076131 that is also located within the 17q12–21 locus^18,19^. Both SNPs are approximately 250 kb away from each other.

The current genotyping study was performed using 4892 Singapore Chinese individuals. Of these, 1938 were already genotyped for tag-SNP rs2941504 in our prior study^14^. These 4892 subjects were all genotyped again in this current study for both tag-SNPs rs2941504 and rs8076131 to avoid any batch variation. In the prior study, the minor allele frequencies (MAFs) of rs2941504 were 0.305 and 0.359 for allergic asthmatic and non-atopic non-asthmatic individuals respectively. For the new participants included in the current study (n = 2954), the MAFs of rs2941504 were 0.339, 0.365, and 0.392 for allergic asthmatic, atopic non-asthmatic, and non-atopic non-asthmatic individuals respectively.

Both rs2941504 and rs8076131 were genotyped in 4892 individuals recruited from the Singapore Chinese population. Genotyping results for both SNPs were tested for and passed the Hardy-Weinberg equilibrium test. In terms of the LD relationship between these 2 SNPs, the R^2^ value as inferred from this current genotyping study is 0.38. As shown in Table 1, the allele “A” of PERLD1 SNP rs2941504, was significantly associated with an increase in the risk of allergic asthma when comparing the diseased cases with atopic non-asthma (p = 0.003, adjusted odds ratio, OR = 1.339), or with non-atopic non-asthma control individuals (p = 6.75 × 10^−6^, adjusted OR = 1.166). The other SNP rs8076131 was also found associated with the disease (p = 7.10 × 10^−5^, and p = 1.85 × 10^−5^, using atopic and non-atopic as control groups respectively, Table 1). As both of these SNPs were disease-associated, a conditioned association test was performed to confirm whether both are independent signals. After conditioning against rs8076131, the association of rs2941504 with allergic asthma remained significant for the comparison between disease and non-atopic non-asthma individuals (p = 0.0365, Table 1). This implies PERLD1 SNP rs2941504 as an independent disease-association signal, as compared to SNP rs8076131.

**Table 1:** Population Genotyping Results of rs2941504 in Singapore Chinese Population.

SNP	Minor/Major Allele	MAF (Ca/Co)	No. of Subjects (Ca/Co)	P	OR	CI (U95-L95)	P_COND_	P_HWE_
**Allergic Asthma vs Non-atopic Control**								
rs2941504	G/A	0.329/0.391	1250/1124	6.75E-06*	1.339	1.179–1.520	0.0365*	1
rs8076131	G/A	0.213/0.272	1250/1124	1.85E-05*	1.362	1.182–1.568	—	0.17
**Allergic Asthma vs Atopic Control**								
rs2941504	G/A	0.329/0.364	1250/2518	0.00370*	1.166	1.051–1.294	0.981	1
rs8076131	G/A	0.213/0.256	1250/2518	7.10E-05*	1.271	1.129–1.431	—	0.17
**Atopy non-asthma vs Non-atopic Control**								
rs2941504	G/A	0.364/0.391	2518/1124	0.0259*	1.126	1.014–1.249	0.0739	1
rs8076131	G/A	0.256/0.272	2518/1124	0.144	1.089	0.971–1.221	—	0.17

*Logistic regression test was used, with adjustment to the test subject’s age and gender. Ca: cases; Co: controls; CI: confidence interval; MAF: minor allele frequency; P: adjusted logistic regression p-value; OR: adjusted odds ratio, using minor allele as the reference category; L95: lower 95% confidence level; U95: upper 95% confidence level; P_COND_: adjusted logistic regression p conditioned on rs8076131 genotype; P_HWE_: Hardy-Weinberg equilibrium p-value for entire test cohort.

The association between rs2941504 and the atopic status (as defined as a positive skin prick response to house dust mites) was also evaluated in the same cohort. For direct association, allele “A” was shown significantly associated with an increased risk of atopy development (p = 0.0259, adjusted OR = 1.126, Table 1). However, this association was not significant after being adjusted for rs8076131. This suggests PERLD1 SNP might specifically be associated with allergic asthma but not with their atopic condition, among Singapore Chinese.

### Sequencing of PERLD1 SNPs

**Figure 1:**
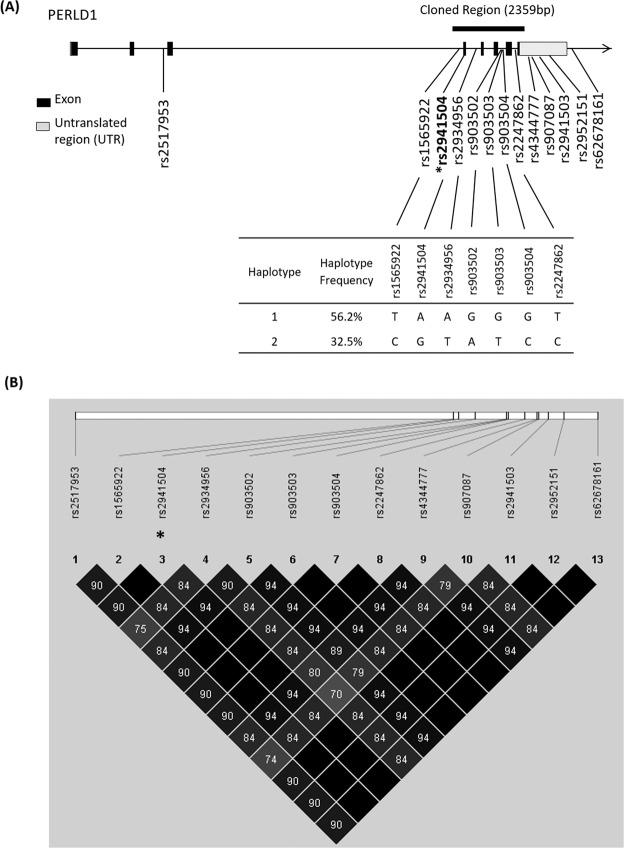
PERLD1 Gene Structure and LD Pattern in Singapore Chinese Population. (**A**) Schematic representation of PERLD1 gene structure with the relative position of 13 common SNPs found in Singapore Chinese. The cloned region for minigene assay was indicated along with frequencies of haplotype used. (**B**) LD pattern for all 13 PERLD1 common SNPs. Significant asthma-associate tag-SNP was indicated with an asterisk (*).

Next, the PERLD1 gene region, including 2 kb upstream and downstream of the gene, was sequenced to localize all SNPs linked with rs2941504 in Singapore Chinese. We recruited 40 individuals from the study population, and sequencing assay has identified 13 common SNPs with MAF more than 5% (Fig. 1A). Among these, 1 is an exonic SNP, 7 are intronic SNPs, 4 are located in the 3′-UTR and 1 is in the downstream region of the gene. LD pattern was constructed and all SNPs were found to be in strong LD (>80%) with rs2941504. This indicates rs2941504 belongs to a set of 13 associated linked SNPs, and these SNPs are all associated with allergic asthma in Singapore Chinese (Fig. 1B).

### *In Vitro* characterizations of PERLD1 SNPs function

The presence of multiple asthma-associated SNPs within the PERLD1 gene region suggests some of these SNPs may functionally influence the gene activity or expression. To investigate this, we first used an *in silico* approach to predict their potential function followed by *in vitro* characterization assays. As summarized in Table 2, exonic SNP rs2941504 was predicted to be synonymous and will not result in a change in the amino acid valine that it codes for. However, using Human Splicing Finder^20^, this SNP and 7 other intronic SNPs were found likely to affect the splicing machinery of PERLD1 transcript, as they are located in exon splicing enhancers (ESE) or silencers (ESS) region of the gene. The other 4 SNPs located in 3′-UTR were ruled out of their possible roles in affecting mRNA stability, as none of them were predicted to affect miRNA binding in this region^21^. The only intergenic SNP rs62678161, located downstream of the gene region, was unlikely to have any functional effect.

**Table 2:** Functional Predictions of 13 PERLD1 Common SNPs Found in Singapore Chinese Population.

SNP rsID	Location	Variation	MAF	Predicted functionality
rs2517953	Intron 2	C > G	0.375	ESE (SRp55, SC35)
rs1565922	Intron 3	T > C	0.400	ESE (SC35, SF2/ASF, SRp55), ESS
rs2941504	Exon 4	T > C	0.400	Synonymous, ESE (9G8, Tra2-β)
rs2934956	Intron 4	A > T	0.412	ESE (9G8, Tra2-β, SC35, SF2/ASF, SRp40)
rs903502	Intron 6	G > A	0.388	ESE (9G8, SF2/ASF), ESS
rs903503	Intron 6	G > T	0.400	ESS
rs903504	Intron 6	G > C	0.400	ESS
rs2247862	Intron 7	T > C	0.400	ESE (SRp55, SF2/ASF), ESS
rs4344777	3′-utr	G > A	0.400	—
rs907087	3′-utr	T > C	0.388	—
rs2941503	3′-utr	A > G	0.400	—
rs2952151	3′-utr	G > A	0.400	—
rs62678161	intergenic	T > C	0.400	—

*Predictions of splice regulation by exonic or intronic SNPs were done using Human Splicing Finder. The proposed Exonic Splicing Enhancer (ESE) or Exonic Splicing Silencer (ESS) was shown, with respective splice regulator proteins listed out in brackets. MiRBase was used for miRNA binding predictions for SNPs within 3′-UTR region. MAF: minor allele frequency.

As 8 out of 13 PERLD1 SNPs were located in predicted ESE or ESS region of the gene, *in vitro* minigene assay was conducted to study differential exon splicing events that might be associated with these SNPs. Approximately 2.4 kb gene region spanning exon 4 to exon 7 of PERLD1 was evaluated, with a small portion of intron 3 (upstream of exon 4) and 3′-UTR also included (Fig. 1A). This region comprises 7 SNPs that form 2 common haplotypes; with major haplotype (Hap1) and minor haplotype (Hap2) having a frequency of 56.2% and 36.5% in the study population respectively (Fig. 1A). SNP rs2517953 was not included as it located in exon 2 which is almost 10 kb away from our current study region. The inclusion of this SNP to the study would exceed the maximum construct size allowed into the expression vector for transfection in this assay.

**Figure 2:**
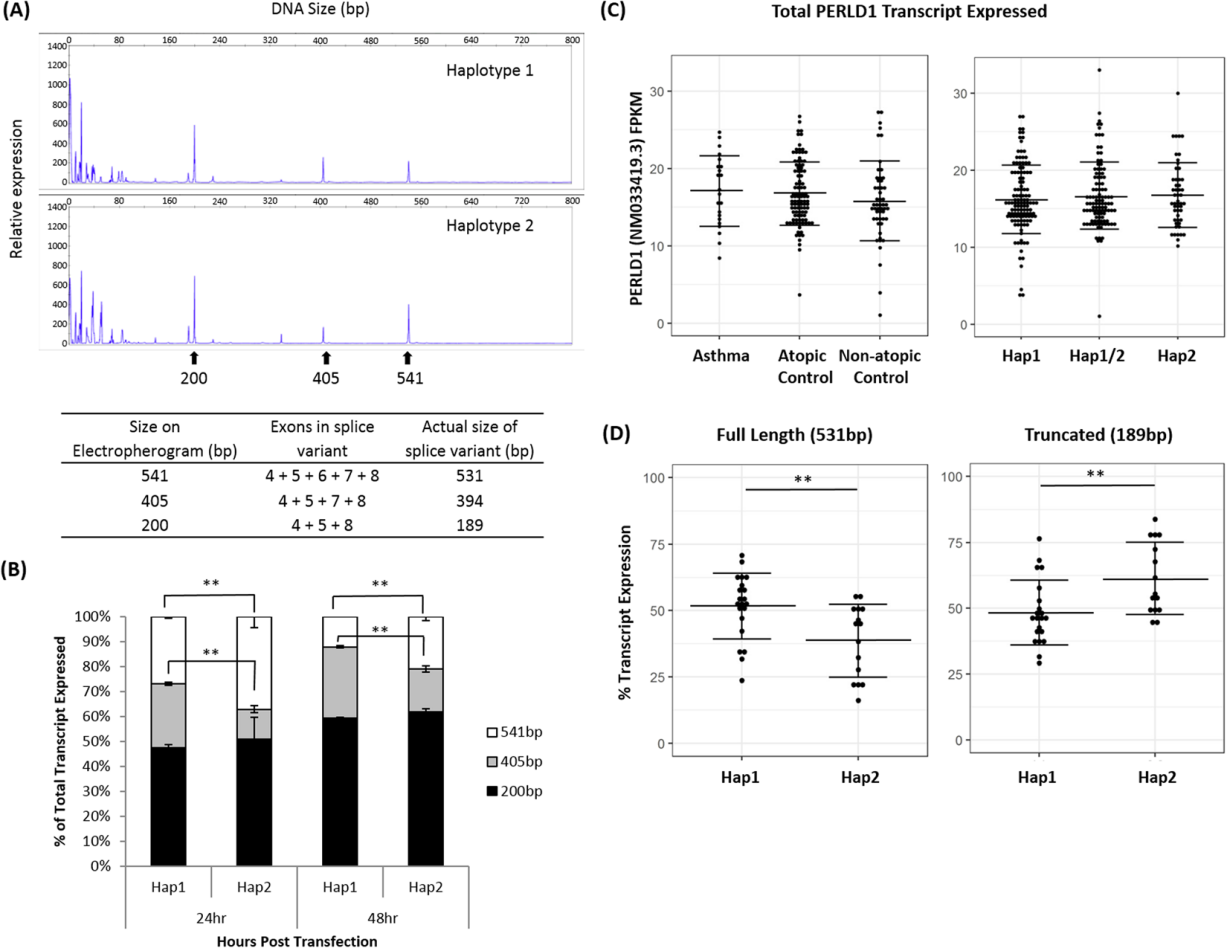
Effect of haplotypes on PERLD1 splice variants. (**A,B**) Fragment analysis of PERLD1 transcripts expressed from HEK293T. (**A**) 24 hours post-transfection with Hap1 or Hap2 of gene constructs. The DNA size of each peak (in bp) was also indicated. Transcript size on the electropherogram and actual size expected for each PERLD1 transcript variant was also summarized. (**B**) Percentage of each transcript variants expressed among total PERLD1 transcripts. PERLD1 transcripts expressed in HEK293T were measured 24 or 48 hours post-transfection. **T-test p-value < 0.01. (**C,D**) Fragment analysis of PERLD1 transcripts expressed in PBMCs from Singapore Chinese human participants. (**C**) Percentage of PERLD1 full length (541 bp) or truncated transcript (200 bp) expressed over total PERLD1 transcripts, compared across PERLD1 haplotypes. **T-test p-value < 0.01. (**D**) Overall PERLD1 transcript expression measured as fragments per Kilobase of transcript per Million mapped reads (FPKM) and separated according to test subject’s phenotype (left) or haplotype (right) group.

**Figure 3:**
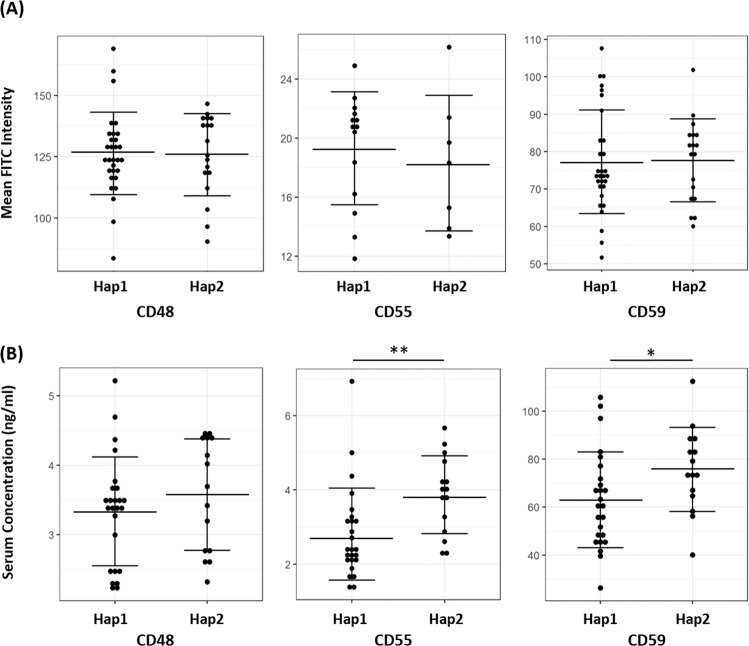
Expressions of Selected GPI-APs in Singapore Chinese Population. (**A**) The surface density of CD48, CD55, and CD59 on T helper cells (gated for CD3+ CD4+ CD8−) was represented as average FITC staining intensity on PBMCs from human subjects. Test subjects were separated according to their PERLD1 haplotype groups. (**B**) The serum concentration of CD48, CD55, and CD59 from participants recruited from the study population. Test subjects were separated according to their PERLD1 haplotype groups. *T-test p-value < 0.05. **T-test p-value < 0.01.

We transfected Hap1 or Hap2 minigene constructs into HEK293T cells separately. The amount and length of transcript expressed were measured at 24 or 48 hours post-transfection by capillary electrophoresis, in the form of fluorescence intensity peak shown in the electropherogram. These peaks were found to be approximately 10 bp longer than the expected length of PERLD1 transcript, as M13 sequence was added to the 5′-end of PERLD1 transcripts to label them with fluorescein amidite (FAM) dye. As shown in Figs. 2A and 3 distinct peaks with transcript length of 541 bp, 405 bp, and 200 bp were expressed from cells transfected with either Hap1 or Hap2 constructs. The 541 bp peak corresponds to a PERLD1 transcript of 531 bp in actual length, which comprises all exons from 4 to 7. The 405 bp peak is due to the absence of exon 6 (137 bp) within PERLD1 transcript, which is 394 bp in actual transcript length. This was further sequence confirmed. Lastly, the 200 bp peak corresponds to a PERLD1 transcript of 189 bp in actual length, with both exon 6 (137 bp) and 7 (205 bp) truncated. Again, these were all sequence confirmed independently.

Quantitative analysis for each PERLD1 splice variant was performed based on peak area measured from the electropherogram. Proportionately, in the 24 hours transfection assay, Hap1 results in a 27% abundance of 541 bp transcripts among total PERLD1 splice variants, and this is significantly lower than that of Hap2, which has 48% abundance (p < 0.01, Fig. 2B). The proportion of the 405 bp transcript was in turn higher for Hap1 (26%) as compared to Hap2 (15%, p < 0.01, Fig. 2B). A similar expression level was observed for 189 bp transcript across both haplotypes. On the other hand, the 48 hours assay also showed similar trends with a significant difference between haplotypes (p < 0.01 for both transcripts of 541 bp and 405 bp, Fig. 2B).

### Expression of PERLD1 splice variant

Having identified the differential splicing event in PERLD1 transcript and its association with PERLD1 haplotype in HEK293T, we next examine if a similar pattern of transcription would be observed from the PBMCs of human subjects *ex vivo*. We recruited human participants homozygous for Hap1 or Hap2 only and assessed PERLD1 splice variant expression using the same capillary electrophoresis approach. In these test subjects, splice variants of 189 bp and 531 bp in length were identified from PBMCs, which corresponds to 2 of the 3 transcripts detected in HEK293T. PERLD1 transcript with only exon 6 truncated (405 bp) was not detected in PBMCs of human subjects. The percentage of 189 bp transcript expressed over the total transcript was found significantly lower in Hap1 than that of Hap2 (p < 0.01, Fig. 3B). Relatively, the same expression ratio for 531 bp PERLD1 transcript was found reduced in Hap2 individuals compared to that of Hap1 (p < 0.01, Fig. 2C).

In addition to the effect on splicing machinery caused by PERLD1 haplotypes, we also confirmed that the overall PERLD1 gene expression remained unchanged between Hap1 and Hap2. A cohort of 297 Chinese individuals was recruited and PBMC expression of PERLD1 transcript (NM033419.3) was assessed through next-generation sequencing. We confirmed the 278 test subjects’ haplotypes through genotyping for rs2941504, and overall PERLD1 expression was shown unchanged across Hap1, Hap2, and Hap1/Hap2 heterozygous group of individuals (Fig. 2D). Besides, for 175 test subjects within this cohort with well-defined asthma-related phenotype, no significant difference in terms of overall PERLD1 expression was observed among subgroups of asthma, atopic non-asthma, and non-atopic non-asthma individuals (Fig. 2D).

### GPI-APs expression

**Figure 4:**
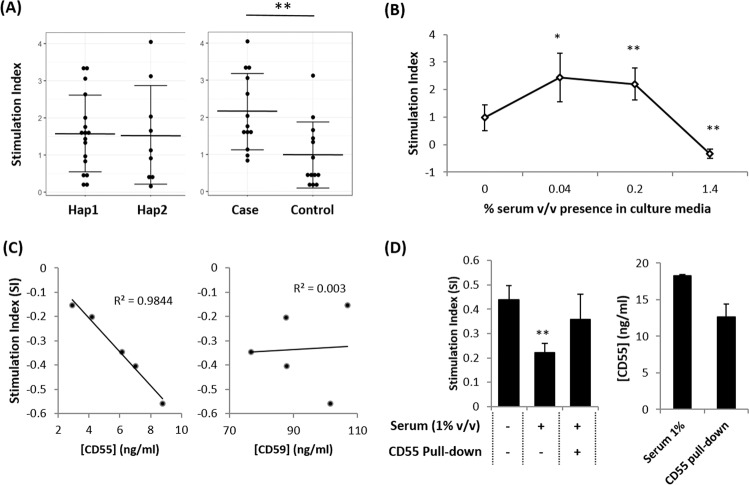
Effect of Serum from Human Subjects towards PBMC Reactivity upon Stimulation. (**A**) Stimulation index (SI) of PBMC from 26 human volunteers from the Singapore Chinese population after phytohaemagglutinin (PHA) stimulation, stratified according to their PERLD1 haplotypes or allergic asthma phenotypes. *T-test p-value < 0.05. **T-test p-value < 0.01. (**B**) Stimulation index (SI) of PBMC from 5 human volunteers (2 asthmatics and 3 non-asthmatics) from the study population under the influence of different v/v percentage of serum in the culturing medium. (**C**) 5 test subjects’ (2 asthmatics and 3 non-asthmatics) serum levels of CD55 or CD59 were plotted against SI under 1.4% serum treatment. The correlation coefficient (R^2^) was indicated in the figure. (**D**) SI of PBMC under influence of 1% v/v serum in the culture medium and the effect of CD55 pull-down treatment. **T-test p-value < 0.01. **(E)** Effect of CD55 pull-down assay on serum concentration of CD55.

Due to the functional involvement of PERLD1 in lipid remodeling of GPI anchors, cellular expressions of several GPI-APs were examined. These include CD48, CD55 and CD59. We extracted PBMCs of 50 human subjects recruited from the Singapore Chinese population and selected only T helper cells (gated for CD3+ CD4+ CD8−, Supplementary Fig. 1) for measurement of surface GPI-APs. As shown in Fig. 4A, all 3 GPI-APs of study interest were successfully detected in the selected cell type and showing average FITC density reading of 133.50, 83.75, and 18.98 for CD48, CD55, and CD59 respectively. Nevertheless, no significant difference was observed between Hap1 and Hap2 individuals for CD48, CD55, and CD59 surface marker reading (Fig. 3A).

Although the current study has shown PERLD1 haplotype might not affect surface expression of tested GPI-APs, the concentration of these proteins in soluble form was found to be correlated with their haplotypes instead. Within the same cohort for GPI-APs surface expression study, we also obtained 40 human participants’ serum samples and measured serum level of soluble CD48, CD55, and CD59. Within test subjects, Hap2 was found to have significantly increased levels of sCD55 and sCD59 in the serum (p < 0.01 and p < 0.05, respectively, Fig. 3B). We also observed a similar trend for elevated serum level of sCD48 associated with Hap2, although the association is not statistically significant (Fig. 3B).

### Soluble GPI-APs inhibition on PBMC proliferation

Next, we investigated whether differential PERLD1 haplotypes correlate with immune cells reactivity. We assayed PBMCs from human participants of the Singapore Chinese cohort (n = 26) for their proliferation rate upon stimulation with PHA. These PBMCs samples were cultured in medium enriched with 10% fetal bovine serum (FBS) to exclude possible influence from the test subject’s serum. As shown in Fig. 4A, there was no significant difference in the stimulation index (SI) of PBMC between PERLD1 haplotypes. We speculated that PERLD1 haplotypes might only influence the proliferation rate of PBMCs through regulating the test subject’s serum sGPI-APs level. Besides, test subjects affected by allergic asthma have shown higher SI of PBMC among allergic asthma individuals (t-test p < 0.01, Fig. 4A). This observation corresponds with the previous findings that PBMC showing distinctive cytokine profile towards stimulation in asthmatic patients, as compared to control subjects.

We then tested our speculation that serum sCD55 and sCD59 levels would influence immune cells reactivity. SI of PBMCs was assessed with the influence of human test subject’s serum. With initial treatment of 0.04% v/v of serum, the SI of PBMC was higher than the untreated (0% serum) counterpart (p < 0.05). This increasing trend of SI was also observed in cells treated with 0.2% v/v serum (p < 0.01 compared to 0% serum-treated PBMC). In contrast to these 2 treatment groups, SI was instead reduced for PBMC treated with 1.4% v/v serum. This reduction was also found to be significantly different from that of 0% serum treated PBMC (p < 0.01, Fig. 4B).

In addition to the reduced SI resulted from 1.4% serum treatment, this reduction was also found associated with GPI-APs concentration within the test subject’s serum. Through further stratification of our serum inhibition assay on PBMC of 5 test subjects, we observed the increasing amount of sCD55 in the serum would result in suppression of PBMC proliferation under stimulation (R^2^ = 0.9844, Fig. 4C). A similar analysis was also performed by comparing serum sCD59 level with SI of PBMC although they were not found to be correlated (R^2^ = 0.003, Fig. 4C).

The inhibitory effect of serum sCD55 on PBMC proliferation was further validated with a partial pull-down of this marker in the serum, using magnetic beads conjugated with CD55 antibody. We used PBMC from several of the individuals studied above, and re-cultured it with the addition of 1% v/v test subject’s serum, or with 0% serum. The SI was observed to be significantly reduced with 1% serum treated PBMC (p < 0.01), as compared to 0% serum treated control. Next, with approximately 31% of sCD55 partially removed from the serum, we managed to rescue the SI of the PBMC back and achieve a level comparable to that of 0% serum treated control (Fig. 4D,E). This suggests sCD55 as a crucial component for serum-mediated suppression of PBMC proliferation.

## Discussion

PERLD1 is involved in the lipid remodeling process of GPI anchors^5^. This remodeling process enables the raft association of GPI-APs onto the cell membrane^22^. As multiple GPI-APs are involved in T-cell signaling^7^, a genetic defect in PERLD1 may influence the signaling process and downstream immune cell functionality. Our group has previously identified an allergic asthma-associated SNP rs2941504 in PERLD1 through the pooled GWAS approach^14^. This current study has shown this association to be independent of another asthma-associated signal, rs8076131, within the same 17q12–21 region. Further to this, we characterized the function of a haplotype group formed by rs2941504 and other linked PERLD1 SNPs that are associated with the disease. Differential PERLD1 haplotypes have resulted in the differential splicing event of the PERLD1 transcript and also correlate with downstream soluble GPI-APs expression and sensitivity of human PBMC towards PHA stimulation. This is the first report that links genetic variants in the PERLD1 gene with immune cell functionality.

Differential splicing event of PERLD1 transcript was shown firstly through *in vitro* minigene assay using mammalian cell models of kidney origin (HEK293T). In addition to this kidney cell model, our group had attempted to demonstrate this differential splicing event in several T-cell models such as Jurkat and E6-1 cell lines. This is due to the functional involvement of PERLD1-modified GPI-APs in T-cell activation^7^. Nevertheless, the PERLD1 transcript was not successfully spliced within the T-cell models tested. We suspected the expression vector used in this current study might not be suitable for these T-cell lines, as more complicated splicing machinery might exist. This was also reflected by the fact that no report on minigene assay was conducted in any other mammalian cell lines, except HEK293T^23,24^. Due to this technical limitation, we thus assessed the differential splicing event of PERLD1 in PBMC samples, using the same capillary electrophoresis approach.

The current study has observed 3 different PERLD1 splice variants in the HEK293T cell model, however, only the full length (531 bp) and truncated (189 bp) PERLD1 transcripts were found expressed in the PBMC of human subjects. Besides, the expression levels of full-length and truncated PERLD1 transcripts were contradictory between these 2 models. These contradictory results may indicate tissue-specific regulation of PERLD1 transcript splicing machinery. We have further assessed the PERLD1 protein expression using western blot of *in vitro* mammalian cell culture however only the full length functional PERLD1 protein was detected. Further, we did not observe differential expression of this PERLD1 protein levels between cells transiently transfected with PERLD1 hap1 and hap2 gene constructs (results not shown). As compared to the HEK293T cell model that is transiently transfected with the PERLD1 gene, the expression patterns for PERLD1 splice variants in PBMC samples are considered more closely related to the actual physiological condition of human immune cells. By assessing the expression of the PERLD1 splice variant in the PBMC samples, Hap2 individuals were found to have an increased proportion of truncated transcript (189 bp) among the total PERLD1 transcript expressed. The loss of exon 6 and 7 from this truncated transcript would translate to a loss of amino acids 186 to 299, in which this region contributes about 35% of the peptide from the PERLD1 protein. Using yeast Per1p protein that is homologous to human PERLD1, it has been shown that the truncated region is required for the proper function of this protein^5^. This implies that the 189 bp PERLD1 splice variant may not be functional, which could reduce the overall PERLD1 activity in the PBMCs. As demonstrated previously using the yeast homolog Per1p protein, a reduction in the PERLD1 function might associate with the defective lipid re-modification process of GPI-anchors^5^.

The fatty acid remodeling process of GPI-anchors is required for proper association of GPI-APs onto the cell membrane^22^. Therefore, the consequence of a defective fatty acid remodeling process could be an increase in the sequestration of GPI-APs into the outer cell compartment. Indeed, this current study has shown Hap2 individuals having elevated serum sCD55 and sCD59 levels as compared to that of Hap1. Following this, a reduction in the PHA-stimulated proliferation rate was observed for PBMCs cultured with 1.4% of these autologous serum samples, although an initial increment of this proliferation rate was observed with 0.04% and 0.2% serum. This initial increment is expected as animal serum has been widely used to enrich culture media for stimulating proliferation and promoting survival in most cell culture study^25^. Also, under treatment with 1.4% serum, the reduction in PHA-stimulated proliferation rate of PBMCs could be due to the presence of sCD55 in the serum. This is supported by our current findings that showed a negative correlation between the PHA-stimulated PBMC proliferation rate and the sCD55 level in the autologous serum. Moreover, by inducing the secretion of serum sCD55 in a mouse model, a prior study has correlated sCD55 level with both inactivation of the complement system and reduction of atopic dermatitis-related symptoms^26^. Studies on mouse models have also shown enhanced T-cell proliferation rate by knocking out CD55 expression on the cell surface, while subsequent knocking out of C3 or Factor D that belongs to the complement activation pathway has restored the T cell response to normal level^11,12^. This implies cell surface CD55 could regulate T cell activity by inhibiting the complement pathway. Based on these prior findings, we speculated serum sCD55 may function similarly as the cell surface CD55 protein to suppress the complement activation pathway and subsequently regulate T-cell immunity.

Another possible mechanism for which serum sCD55 could suppress the immune cell activity involves the activation pathway of CD55 receptor by CD95. It has been shown that CD55 on the CD4+ T-cell surface can be activated by CD95, while both of them subsequently co-stimulate this immune cell upon activation^13^. Therefore, as observed in this current study, the abundance of sCD55 in test subjects’ serum may act competitively to bind CD95 ligand and inhibit the signaling pathway. This would then result in the lower sensitivity of T-cells towards stimulation. Overall, both complement-dependent and CD95-dependent signaling pathways are possible explanations of how sCD55 may reduce the proliferation rate of immune cells. Further study on the activation process of these 2 pathways using PBMC samples is therefore required to confirm these speculations.

The activation and proliferation of human T-cells is an important process during the pathogenesis of asthma^2,3^. This current study has associated a functional PERLD1 haplotype with the proliferation capability of human lymphocytes and other immune cells upon stimulation. Individuals carry PERLD1 Hap2 tend to have increased serum levels of several GPI-APs, including sCD55 and sCD59. Among these, sCD55 was proven to be potent in reducing the proliferative capability of immune cells towards PHA stimulation. Hap2 is formed by the minor allele of tag-SNP rs2941504, which has been proven to associate with reduced allergic asthma risk in the Singapore Chinese population. Therefore, the protective attributes of rs2941504 minor allele might be contributed by its role in immune cell function, thus reducing susceptibility to allergic asthma.

## Materials and Methods

### Ethics statement

Human subjects recruited in this current study belong to a part of ongoing epidemiology study, with approval granted from Institutional Review Boards (IRB) in the National University of Singapore (NUS-IRB Ref-Code: B-10-343 and B-14-150 for genomic DNA and blood collection separately; NUS-IRB Ref-Code: 07–023, 09–256, 10–445, and 13–075, for the large scale epidemiology and genetics study) and the Institutional Review Board of the National Healthcare Group Domain Specific Review Board - B/04/055). Recruitment of a separate cohort of human subjects from Malaysia also obtained approval from Scientific and Ethical Review Committee (SERC) in the University of Tunku Abdul Rahman (UTAR), Malaysia (UTAR-SERC, Ref code: U/SERC/03/2016). All recruitment procedures were performed in concordance with the Helsinki Declaration. Written and informed consents were obtained from all participated individuals, with parental/guardian consent obtained additionally for individuals below 21 years old.

### Human subject recruitment

The population genotyping cohort was recruited from the National University of Singapore, with Chinese ethnicity confirmed through survey questionnaires, and also through previously performed principal component analysis^27^. The demographics of the participants are included in Table 3. Among these 4892 individuals, there are 1938 individuals already genotyped for rs2941504 using the allele-specific PCR approach in our previous study^14^. These 4892 subjects were all genotyped again in this current study for both tag-SNPs rs2941504 and rs8076131 using the genotyping array approach to avoid any batch variation. Subjects with allergic asthma were defined as having the disease diagnosed in a local clinic or hospitals, and also having an atopic condition. Subjects having a positive skin prick test (SPT) reaction towards one of 2 common house dust mite (HDM) species (Blomia tropicalis or Dermatophagoides pteronyssinus) are considered atopic while having no positive SPT reaction towards any HDM are considered non-atopic. The SPT was performed during the participant recruitment process.

**Table 3:** Demographics of Singapore Chinese Population for Genotyping Study.

	Allergic Asthma	Atopic Non-asthma	Non-atopic Non-asthma	Total
Total n	1250	2518	1124	4892
Gender: Male (n, %)	700 (56.00)	1148 (45.59)	324 (28.83)	2172 (44.40)
Female (n, %)	548 (43.84)	1367 (54.29)	799 (71.09)	2714 (55.48)
Missing (n, %)	2 (0.001)	3 (0.001)	1 (0.001)	6 (0.001)
Age (Year, Mean ± 1 SD)	20.60 ± 4.73	21.81 ± 3.72	22.20 ± 4.95	21.59 ± 4.34

### Genotyping

Participants’ genomic DNA (gDNA) samples for genotyping assay were extracted from mouthwash samples. The extraction was performed using Axygen® AxyPrep™ Multisource Genomic Miniprep DNA kit, according to the manufacturer’s protocol. The concentration of extracted gDNA was measured in triplicates using NanoDrop 2000 spectrophotometer (Thermo Scientific, Singapore). Genotyping of 4895 gDNA samples was performed using 4 GWAS arrays, namely Infinium OmniZhongHua-8 v1.3 BeadChip platform, Illumina HumanHap 550 k BeadChip version 3, InfiniumOmni2–5Exome, and Infinium Global Screening Array. Haplotype phasing and imputations of the data were performed using the IMPUTE2 program. Results from all 4 arrays were compiled subsequently for downstream analysis of the SNP-disease association.

### Sequencing and functional predictions of PERLD1 gene variants

PERLD1 gene region, including 2 kb upstream and 1 kb downstream regions of the gene, was sequenced through Sanger sequencing approach^28^. A total of 40 human volunteers were recruited from Singapore Chinese, with their buccal cells collected for DNA extraction. All single nucleotide polymorphisms (SNPs) identified was reconfirmed through re-sequencing of the opposite strand. Subsequently, these SNPs were compared with the SNP database available in NCBI [http://www.ncbi.nlm.nih.gov/SNP/] and Ensemble databases [http://www.ensembl.org /index.html] for the acquisition of SNP rs ID. The r^2^ value between SNPs and linkage disequilibrium pattern were calculated and drawn using software Haploview® vers4.2 [http://www.broadinstitute.org/haploview]. The *in silico* functional predictions of the identified SNPs were predicted using the F-SNP database [http://compbio.cs.queensu.ca/F-SNP/]^29^. Splicing regulatory sites were further assessed using Human Splicing Finder^20^. SNPs located within 3′-UTR of the gene were tested for potential miRNA binding sites using miRBase [http://www.mirbase.org/], using recommended approach^21^.

### *In vitro* minigene assay in HEK293T

Human embryonic kidney cells (HEK293T) were purchased from the American Type Culture Collection (ATCC). The cells were grown in RPMI-1640 medium (Sigma-Aldrich Co.), supplemented with 10% fetal bovine serum, 2 g/L sodium bicarbonate and 2 mmol/L L-glutamine. The cells were incubated in 37 °C humidified incubator with a 5% CO_2_ environment. Minigene assay was conducted using the mammalian expression vector pcDNA3.1( + ) (Invitrogen). The desired PERLD1 gene insert was PCR amplified using forward primers “GAGTAACTAGGGAGCTTGGCTATACCG” and reverse primers “AGAACAGACTCCAAGGCTGGTGAGG”. The plasmid and PERLD1 insert were double-digested at NotI and EcoRV restriction enzyme sites and fused using T4 DNA ligase. Transient transfection of expression plasmids into HEK293T was done using Lipofectamine 2000 (L2000, Invitrogen, Singapore) at a 1ul L2000 to 1ug plasmid ratio. The cells were harvested 24 or 48 hours post-transfection for downstream analysis. RNA was immediately extracted from the harvested cells using E.Z.N.A.® Total RNA Kit I (Omega Bio-Tek Store) and converted to cDNA using ProtoScript® First Strand cDNA Synthesis Kit (New England Biolabs) for subsequent analysis of transcript expression, according to manufacturers’ recommended protocols.

### Fragment analysis of PERLD1 splice variants

Capillary electrophoresis was done for PCR amplified PERLD1 cDNA transcripts extracted from HEK293T of the minigene assay. PERLD1 transcripts were amplified at region spanning exon 4 to 7, using forward primers “GTGTCCCTCAATGCATGGTTCTGGT” and reverse primers “GTCCAGCTTGAACTTGTCCTCTGATTC”. Additional M13 tag sequence “CACGACGTTGTAAAACGAC” was added to the 5′-end of forward primer to label the product with Fluorescein amidite (FAM) dye. The FAM-labeled product was analyzed for its DNA fragment length using Applied Biosystems 3130xl Genetic Analyzer, according to the manufacturer instructions. A total of 50 Singapore Chinese were recruited subsequently, for the measurement of PERLD1 splice variants expression in peripheral blood mononuclear cells (PBMC). Blood samples were collected from the test subjects, with their PBMC extracted using Ficoll-Hypaque density-gradient centrifugation. RNA samples were extracted using E.Z.N.A.® Total RNA Kit from Omega Bio-tek Inc., according to the manufacturer’s protocols. Conversion to cDNA was performed for subsequent analysis of transcript expression using ProtoScript® First Strand cDNA Synthesis Kit (New England Biolabs). Capillary electrophoresis was done subsequently with the same method as that of the minigene assay using HEK293T.

### Next-generation sequencing of PERLD1 mRNA transcript

The overall expression of PERLD1 mRNA transcript was measured through next generation sequencing. A total of 297 PBMC samples were collected from Malaysia Chinese individuals in the University of Tunku Abdul Rahman (UTAR), Malaysia and the National University of Singapore. Total RNA was extracted using E.Z.N.A.® Total RNA Kit from Omega Bio-tek Inc., according to the manufacturer’s protocol. For library construction, mRNA was first enriched using oligo(dT) beads, and NEBNext Ultra RNA Library Prep Kit was used. Sequencing was then performed using Illumina NovaSeq 6000 system. Mapping of raw sequence to human genomic sequence (NCBI GRCh38 Assembly) was done using TopHat version 2.1.1. Fragments per Kilobase of transcript per Million mapped reads (FPKM) for ADRB2 transcript (NM000024.5) was calculated using Cufflinks version 2.2.1.

### Flow cytometry analysis of PBMC

T helper cell surface expressions of 3 different GPI-APs, namely CD48, CD55, and CD59, were assayed on 36 recruited Singapore Chinese individuals. PBMCs were first isolated from these participants by Ficoll-Hypaque density-gradient centrifugation approach and used as a source of T helper cells. The isolated PBMC were stained concurrently using 4 different fluorescent stained antibodies: CD3-APC for the selection of T-cells, CD4-Cy5 for the selection of T-helper cells, CD8-Cy7 for selection against cytotoxic T-cells, and either one of CD48-FITC, CD55-FITC or CD59-FITC to stain for the corresponding GPI-AP. The antibodies were all purchased from Abcam. Flow cytometry was performed using CyAn™ ADP Analyzer (Beckman Coulter) according to the manufacturer’s instruction manuals. Within each PBMCs sample, lymphocytes were firstly gated according to their expected size (forward scatter) and low granularity (side scatter). Subsequently, the cells were gated to include only T helper cells (CD3+ CD4+ CD8−) according to the gating threshold determined by PBMCs single stained with either CD3-APC, CD4-Cy5, or CD8-Cy7 antibody (Supplementary Fig. 1). Lastly, the mean density of selected GPI-AP was determined based on the mean intensity of FITC staining on T helper cells. All measurements were performed in triplicates.

### Measurement of serum GPI-Aps

Serum concentrations of 3 GPI-APs (CD48, CD55, and CD59) were measured in 40 human volunteers from the Singapore Chinese population. Commercially available Enzyme-linked Immunosorbent Assay (ELISA) kits were exploited for this purpose. Human CD48 and CD55 ELISA kits were purchased from Sino Biological Inc. Cusabio Biotech Co. Ltd, while human CD59 ELISA kit was purchased from USCN Life Science Inc. Wuhan. All assays were conducted according to the manufacturer’s instructions. Samples were diluted in 1xPBS prior measurement for interpolation within the assay standard curve range. Measurements were done in triplicates.

### PBMC stimulation assay

PBMCs were extracted using Ficoll-Hypaque density-gradient centrifugation, as mentioned in the previous section. Immediately after extraction, PBMCs were seeded at 0.1 million cells per well in a 96-well plate and were given a phytohaemagglutinin (PHA, Sigma-Aldrich) challenge at a concentration of 0.1 μg/ml. The cells were cultured with 100 μl RPMI-1640 (Sigma-Aldrich) and with either test subject’s serum or fetal bovine serum. Incubation was done in 37 °C humidified incubator of 5% CO_2_ environment for 5 days. WST-1 reagent (Roche) was then added to the culture medium and incubated for 4 hours to assay optical density (OD). The OD was taken using the Tecan Sunrise microplate reader at 450 nm wavelength absorbance, with a reference wavelength at 650 nm. Stimulation index was calculated by dividing the increased OD after PHA stimulation with the OD of PBMC without PHA stimulation, as shown in the formula below:

$${\rm{Stimulation}}\,{\rm{Index}}=({{\rm{OD}}}_{{\rm{with}}{\rm{PHA}}{\rm{with}}{\rm{serum}}}-{{\rm{OD}}}_{{\rm{no}}{\rm{PHA}}{\rm{no}}{\rm{serum}}})/({{\rm{OD}}}_{{\rm{no}}{\rm{PHA}}{\rm{no}}{\rm{serum}}})$$

For CD55 pull-down assay, the anti-CD55 antibody (Abcam) was conjugated with Dynabeads® Antibody Coupling Kit magnetic beads at a ratio of 5 μg antibodies to 1 mg magnetic beads and incubated at 4 °C on a rotator for 16 hours. Magnetic beads conjugated with an anti-CD55 antibody were then used to pull down its respective proteins by adding 2 mg of beads to 50 μl of test subject’s serum sample for 1 hr. Serum with their CD55 partially removed was then tested for pull-down efficiency using ELISA kit as described above. The serum was also used for the PBMC stimulation assay.

## Supplementary information

Supplementary Figure 1.

## References

[1] The Global Asthma Report 2014, http://www.globalasthmareport.org/resources/Global_Asthma_Report_2014.pdf (2014).

[2] Locksley RM (2010). Asthma and allergic inflammation. Cell.

[3] Holgate ST (2008). Pathogenesis of asthma. Clin Exp Allergy.

[4] Cho JL (2016). Allergic asthma is distinguished by sensitivity of allergen-specific CD4+ T cells and airway structural cells to type 2 inflammation. Sci Transl Med.

[5] Fujita M, Umemura M, Yoko-o T, Jigami Y (2006). PER1 is required for GPI-phospholipase A2 activity and involved in lipid remodeling of GPI-anchored proteins. Mol Biol Cell.

[6] Kinoshita T, Fujita M, Maeda Y (2008). Biosynthesis, remodelling and functions of mammalian GPI-anchored proteins: recent progress. J Biochem.

[7] Loertscher R, Lavery P (2002). The role of glycosyl phosphatidyl inositol (GPI)-anchored cell surface proteins in T-cell activation. Transpl Immunol.

[8] Munitz A, Bachelet I, Finkelman FD, Rothenberg ME, Levi-Schaffer F (2007). CD48 is critically involved in allergic eosinophilic airway inflammation. Am J Respir Crit Care Med.

[9] Muhammad A (2009). Sequential cooperation of CD2 and CD48 in the buildup of the early TCR signalosome. J Immunol.

[10] Deckert M, Ticchioni M, Mari B, Mary D, Bernard A (1995). The glycosylphosphatidylinositol-anchored CD59 protein stimulates both T cell receptor zeta/ZAP-70-dependent and -independent signaling pathways in T cells. Eur J Immunol.

[11] Liu J (2005). The complement inhibitory protein DAF (CD55) suppresses T cell immunity *in vivo*. J Exp Med.

[12] Heeger PS (2005). Decay-accelerating factor modulates induction of T cell immunity. J Exp Med.

[13] Capasso M (2006). Costimulation via CD55 on human CD4+ T cells mediated by CD97. J Immunol.

[14] Anantharaman R (2011). Genome-wide association study identifies PERLD1 as asthma candidate gene. BMC Med Genet.

[15] Halapi E (2010). A sequence variant on 17q21 is associated with age at onset and severity of asthma. Eur J Hum Genet.

[16] Dixon AL (2007). A genome-wide association study of global gene expression. Nat Genet.

[17] Nicodemus-Johnson J (2016). DNA methylation in lung cells is associated with asthma endotypes and genetic risk. JCI Insight.

[18] Andiappan AK (2016). Functional variants of 17q12-21 are associated with allergic asthma but not allergic rhinitis. J Allergy Clin Immunol.

[19] Moffatt MF (2007). Genetic variants regulating ORMDL3 expression contribute to the risk of childhood asthma. Nature.

[20] Desmet FO (2009). Human Splicing Finder: an online bioinformatics tool to predict splicing signals. Nucleic Acids Res.

[21] Griffiths-Jones S, Saini HK, van Dongen S, Enright A (2008). J. miRBase: tools for microRNA genomics. Nucleic Acids Res.

[22] Maeda Y (2007). Fatty acid remodeling of GPI-anchored proteins is required for their raft association. Mol Biol Cell.

[23] Homolova K (2010). The deep intronic c.903+469T>C mutation in the MTRR gene creates an SF2/ASF binding exonic splicing enhancer, which leads to pseudoexon activation and causes the cblE type of homocystinuria. Hum Mutat.

[24] Boulling A, Le Gac G, Dujardin G, Chen JM, Ferec C (2010). The c.1275A>G putative chronic pancreatitis-associated synonymous polymorphism in the glycoprotein 2 (GP2) gene decreases exon 9 inclusion. Mol Genet Metab.

[25] Brunner D (2010). Serum-free cell culture: the serum-free media interactive online database. ALTEX.

[26] Kim Yenny, Lee Youn-Woo, Kim Hangeun, Chung Dae Kyun (2019). Bee Venom Alleviates Atopic Dermatitis Symptoms through the Upregulation of Decay-Accelerating Factor (DAF/CD55). Toxins.

[27] Andiappan AK, Anantharaman R, Nilkanth PP, Wang de Y, Chew FT (2010). Evaluating the transferability of Hapmap SNPs to a Singapore Chinese population. BMC Genet.

[28] Sanger F, Nicklen S, Coulson AR (1977). DNA sequencing with chain-terminating inhibitors. Proc Natl Acad Sci USA.

[29] Lee PH, Shatkay H (2008). F-SNP: computationally predicted functional SNPs for disease association studies. Nucleic Acids Res.

