# Supplementary material for: The Asthma-associated PER1-like domain-containing protein 1 (PERLD1) Haplotype Influences Soluble Glycosylphosphatidylinositol Anchor Protein (sGPI-AP) Levels in Serum and Immune Cell Proliferation
Source: Sci Rep. 2020 Jan 20;10:715. doi: 10.1038/s41598-020-57592-9 (PMC6970992; doi:10.1038/s41598-020-57592-9)
Supplement: Supplementary file 1 — Supplementary Figure 1. [file 41598_2020_57592_MOESM1_ESM.pdf]

Title: The Asthma-associated PER1-like domain-containing protein 1 (PERLD1) Haplotype Influences Soluble Glycosylphosphatidylinositol Anchor Protein (sGPI-AP) Levels in Serum and Immune Cell Proliferation

Yang Yie Sio[1], Ramani Anantharaman[1], Sean Qiu En Lee[1], Sri Anusha Matta[1], Yu Ting Ng[1], Fook Tim Chew[1*]

[1]Department of Biological Sciences, National University of Singapore, Singapore

[*]Corresponding author:

Fook Tim Chew, PhD

Associate Professor, Department of Biological Sciences,

Faculty of Science, National University of Singapore,

Allergy and Molecular Immunology Laboratory,

Lee Hiok Kwee Functional Genomics Laboratories,

Block S2, Level 5, 14 Science Drive 4,

Lower Kent Ridge Road, Singapore 117543.

Phone: +65 65161685

Email: dbscft@nus.edu.sg

Supplementary Data

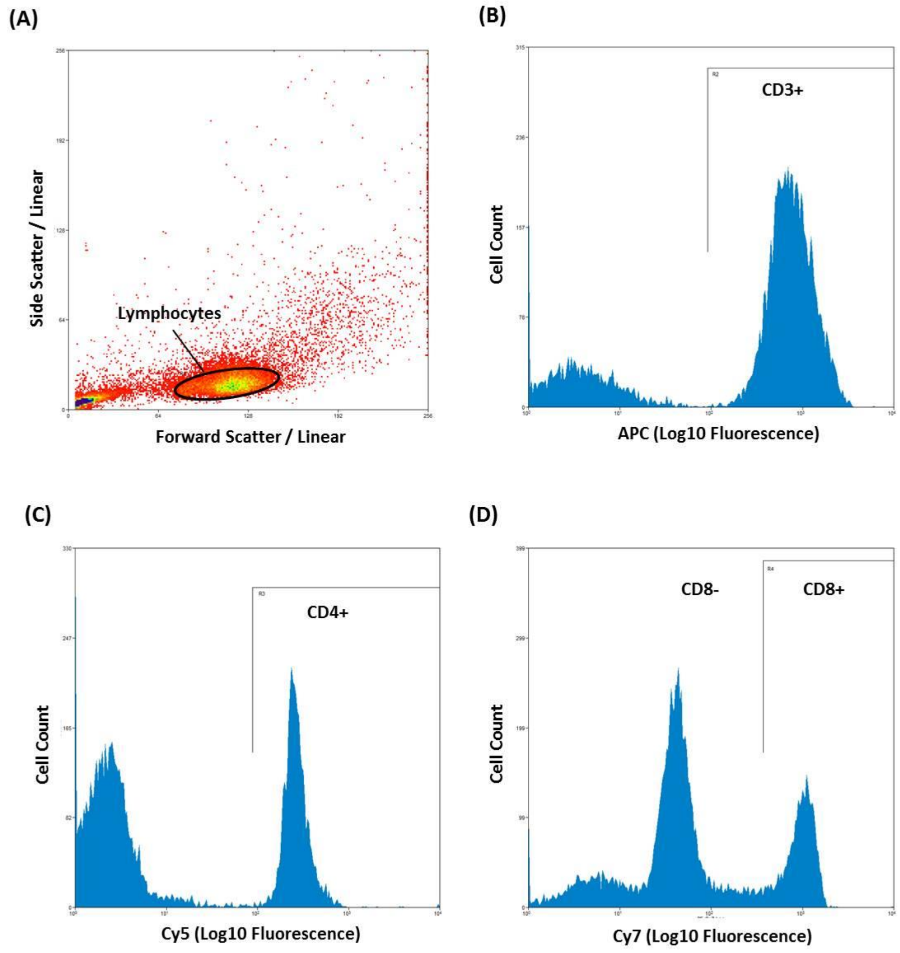

Supplementary Figure 1. Sequential Gating Strategy for Identification of CD3+ CD4+ CD8- T Helper Cells. Optimization of flow cytometry analysis was performed on single antibody stained PBMCs samples for determination of gating threshold. **(A)** Gating to include lymphocytes was based on forward and side scatter, as indicated by a circle in the figure. Subsequent gating optimization was done for lymphocyte cells stained with **(B)** CD3 antibody conjugated with APC, **(C)** CD4 antibody conjugated with Cy5, and **(D)** CD8 antibody conjugated with Cy7.
